# Ambient Air Pollution and Newborn Size and Adiposity at Birth: Differences by Maternal Ethnicity (the Born in Bradford Study Cohort)

**DOI:** 10.1289/ehp.1408675

**Published:** 2015-05-15

**Authors:** Anna Schembari, Kees de Hoogh, Marie Pedersen, Payam Dadvand, David Martinez, Gerard Hoek, Emily S. Petherick, John Wright, Mark J. Nieuwenhuijsen

**Affiliations:** 1 Centre for Research in Environmental Epidemiology (CREAL), Barcelona, Spain; 2 CIBER Epidemiología y Salud Pública (CIBERESP), Madrid, Spain; 3 Universitat Pompeu Fabra, Barcelona, Spain; 4 Department of Epidemiology and Public Health, Swiss Tropical and Public Health Institute, Basel, Switzerland; 5 University of Basel, Basel, Switzerland; 6 Team of Environmental Epidemiology Applied to Reproduction and Respiratory Health, Institute Albert Bonniot, National Institute of Health and Medical Research (INSERM), U823, Grenoble, France; 7 Division of Environmental Epidemiology, Institute for Risk Assessment Sciences, Utrecht University, Utrecht, the Netherlands; 8 Bradford Institute for Health Research, Bradford Teaching Hospitals NHS Trust, Bradford, United Kingdom; 9 School of Health Studies, University of Bradford, Bradford, United Kingdom

## Abstract

**Background:**

Exposure to ambient air pollution has been associated with reduced size of newborns; however, the modifying effect of maternal ethnicity remains little explored among South Asians.

**Objectives:**

We investigated ethnic differences in the association between ambient air pollution and newborn’s size.

**Method:**

Pregnant women were recruited between 2007 and 2010 for the Born in Bradford cohort study, in England. Exposures to particulate matter (≤ 10 μm, PM_10_; ≤ 2.5 μm, PM_2.5_), PM_2.5_ absorbance, and nitrogen oxides (NO_x_, NO_2_) were estimated using land-use regressions models. Using multivariate linear regression models, we evaluated effect modification by maternal ethnicity (“white British” or “Pakistani origin,” self-reported) on the associations of air pollution and birth weight, head circumference, and triceps and subscapular skinfold thickness.

**Results:**

A 5-μg/m^3^ increase in mean third trimester PM_2.5_ was associated with significantly lower birth weight and smaller head circumference in children of white British mothers (–43 g; 95% CI: –76, –10 and –0.28 cm; 95% CI: –0.39, –0.17, respectively), but not in children of Pakistani origin (9 g; 95% CI: –17, 35 and –0.08 cm; 95% CI: –0.17, 0.01, respectively) (*p*_int_ = 0.03 and < 0.001). In contrast, PM_2.5_ was associated with significantly larger triceps and subscapular skinfold thicknesses in children of Pakistani origin (0.17 mm; 95% CI: 0.08, 0.25 and 0.21 mm; 95% CI: 0.12, 0.29, respectively), but not in white British children (–0.02 mm; 95% CI: –0.14, 0.01 and 0.06 mm; 95% CI: –0.06, 0.18, respectively) (*p*_int_ = 0.06 and 0.11). Patterns of associations for PM_10_ and PM_2.5_ absorbance according to ethnicity were similar to those for PM_2.5_, but associations of the outcomes with NO_2_ and NO_x_ were mostly nonsignificant in both ethnic groups.

**Conclusions:**

Our results suggest that associations of ambient PM exposures with newborn size and adiposity differ between white British and Pakistani origin infants.

**Citation:**

Schembari A, de Hoogh K, Pedersen M, Dadvand P, Martinez D, Hoek G, Petherick ES, Wright J, Nieuwenhuijsen MJ. 2015. Ambient air pollution and newborn size and adiposity at birth: differences by maternal ethnicity (the Born in Bradford study cohort). Environ Health Perspect 123:1208–1215; http://dx.doi.org/10.1289/ehp.1408675

## Introduction

Ambient airborne particulate matter (PM) is one of the leading preventable threats to global health (Lim et al. 2012). Evidence of the association of ambient air pollution and restricted fetal growth—expressed as low birth weight (< 2,500 g), small for gestational age, and reduced birth weight—as a continuous measure is growing (Pedersen et al. 2013), but results are heterogeneous across studies (Dadvand et al. 2013; Glinianaia et al. 2004; Stieb et al. 2012). Differences in study design, sample size, population characteristics, adjustment for confounders, air pollution measurements, and exposure assessment techniques as well as the lack of knowledge on the exact biological mechanism are all likely to contribute to the observed heterogeneity of findings (Ritz and Wilhelm 2008; Slama et al. 2008; Woodruff et al. 2009). Although recent studies have successfully overcome issues related to adjustment for confounders, sample size, study design, and exposure assessment (Dadvand et al. 2013; Pedersen et al. 2013), as far as we are aware there is no study to date that assesses the impact of maternal exposure to air pollution on newborn’s fat mass. Animal studies suggest that gestational exposure to PM_2.5_ increases the predisposition to insulin resistance and to thicker adipose tissues (Bolton et al. 2012). Maternal smoking during pregnancy has been associated with reduced offspring size at birth (weight, length, and head circumference), but not with smaller skinfold thickness (Bernstein et al. 2000; D’Souza et al. 1981; Luciano et al. 1998).

In newborns, measurements of upper body skinfold thickness may provide an estimate of the total body fat of the infant (Farmer 1985). Postnatal fat accumulation occurs predominantly in the extremities; hence triceps (upper arm) skinfold thickness provides an indication of the peripheral body fat mass, and subscapular (upper back) skinfold thickness reflects the subcutaneus/visceral fat (Ketel et al. 2007; Snijder et al. 2006). Subcutaneous/visceral fat mass has increasingly been related to the pathogenesis of insulin resistance and other precursors of cardiovascular disease (Patel and Abate 2013; Sniderman et al. 2007). Growth trajectory throughout childhood, including skinfold thickness during infancy and childhood, has been extensively studied in England (Tanner and Whitehouse 1962) and in India (Bansal et al. 2008; Krishnaveni et al. 2005; Yajnik et al. 2002, 2003).

Infants of South Asian origin have a higher prevalence of low birth weight than White Europeans [United Nations Children’s Fund, World Health Organization (UNICEF, WHO) 2004], and the difference seems to be independent of socioeconomic status (Margetts et al. 2002). The total body fat volume of South Asian newborns is similar to the one of white British newborns, but their mean birth weight is 200 g lower (West et al. 2013; Yajnik et al. 2003). During childhood and adolescence, South Asians have relatively greater subscapular skinfolds than white British (Bansal et al. 2008; Krishnaveni et al. 2005), which may lead to disproportionate subcutaneous adiposity for a given body mass index (BMI) (Snijder et al. 2006) and increased risks of adverse cardiometabolic outcomes including obesity, insulin resistance, diabetes, and cardiovascular disease in adulthood (Misra and Khurana 2009).

Differences in newborn body composition and the subsequent increased risk of adverse cardiometabolic outcomes has been linked to both newborn’s size and prenatal exposure to ambient air pollution (Thiering et al. 2013).

In the present study, we examined the association of prenatal exposure to ambient air pollution with mean birth weight and head circumference in an ethnically mixed birth cohort population from Bradford, England. Effect modification by maternal ethnicity was examined by comparison of the associations between the two main ethnic groups: white British and Pakistani-origin populations. We also report here the associations between prenatal exposure to ambient air pollution and neonatal adiposity using triceps and subscapular skinfold thickness. Finally, we examined the influence of adjusting for neonatal skinfold thickness on associations between air pollutants and birth weight.

## Methods

*Study population.* Born in Bradford (BiB) is a longitudinal multi-ethnic birth cohort (Wright et al. 2013). Bradford is a city located in the North of England, with a population of approximately half a million in 2011. A high level of socioeconomic deprivation and ethnic diversity characterizes the Bradford city population. There is one maternity unit in Bradford. It is located at Bradford Royal Infirmary, and approximately half of the births are of women with South Asian origin, the majority of whom are Pakistani. Between March 2007 and November 2010, around 80% of the women seeking antenatal care agreed to participate in the BiB study. Participating women completed an interviewer-administered questionnaire around week 26–28 of gestation, from which maternal ethnicity (“white British” and “Pakistani origin”) and residential addresses were derived. Standardized questions were developed using the United Kingdom Office of National Statistics Guidance (West et al. 2013). Although we did not formally assess this issue, very few Pakistani origin women were expected to marry outside their ethnic groups, whereas the proportion of white British women marrying outside their ethnic group could be higher (National Statistics 2005).

Of the 11,396 pregnant women who completed the baseline questionnaires, we excluded 62 children stillbirths, 142 multiple pregnancies, 345 children with missing data on child’s birth weight or gestational age at delivery, 49 women with missing data on ethnicity, and 121 women with missing exposure data; 1,616 infants whose maternal ethnic origin was not white British or Pakistani origin were also excluded. Thus, 9,067 children were included in the analyses: of those, 4,189 (46%) were white British and 4,878 (54%) were of Pakistani origin.

The participants gave informed consent for the data collection and ethical approval for the data collection was granted by Bradford Research Ethics Committee (Ref 07/H1302/112).

*Outcome measurement.* Birth weight (grams) recorded immediately following the delivery (*n* = 9,067) was obtained from the medical records. Head circumference (centimeters; *n* = 8,311), triceps skinfold thickness (millimeters; *n* = 6,188), and subscapular skinfold thickness (millimeters; *n* = 6,169) of the offspring were measured by trained hospital personal within the first 24–72 hr after delivery (West et al. 2013). Skinfold measurements were measured on the left upper arm (triceps) and the left shoulder (subscapular) using Harpenden Calipers (Holtain Ltd) according to standard protocols (Tanner and Whitehouse 1962).

*Exposure assessment.* Exposures to particulate matter (≤ 10 μm, PM_10_; ≤ 2.5 μm, PM_2.5_; PM_2.5_ absorbance) and oxides of nitrogen (NO_x_, NO_2_) were estimated using land-use regression (LUR) models and measurements developed for the European Study of Cohorts for Air Pollution Effects (ESCAPE), as described in detail elsewhere (Beelen et al. 2013; Cyrys et al. 2012; Eeftens et al. 2012). In brief, NO_x_ and NO_2_ were measured during three 2-week campaigns during summer, winter, and an intermediate season within 1 year (2009) in 41 monitoring sites at a combination of traffic, urban, and rural background locations across Bradford. The sites were selected to represent spatial variation of air pollution in the residential areas of the participants. PM_10_, PM_2.5_, and PM_2.5_ absorbance were measured in 20 sites across the Thames Valley (southeast England). A number of geographic information system variables on traffic characteristics, land use, population density, and topography were used to model the measured air pollutants, thus to predict the spatial distribution of exposure to mean levels during 2010. To address the temporal variations related to pregnancies occurring during 2006–2011, we adjusted the LUR spatial annual exposure estimates combining the available daily data of NO_2_, PM_10_, and PM_2.5_ from the background routine monitors located in the cities of Bradford and Leeds. Leeds is at 10 km east of Bradford. Then, the daily spatiotemporal estimates were calculated as the spatial exposure estimation multiplied by the ratio between the daily NO_2_, PM_10_, or PM_2.5_ concentrations at the background routine monitoring station and its annual 2010 average, as described in detail by Pedersen et al. (2013) and by Schembari et al. (2014). Daily spatiotemporal estimates were averaged over the pregnancy period defined above to obtain the final exposures. We further applied the NO_2_ daily temporal adjustment to NO_x_ and PM_2.5_ absorbance spatial estimates because of their high correlation (Cyrys et al. 2003). We assumed that the spatial distribution of pollutants in the city and their determinants remained constant over the study period (Eeftens et al. 2011).

Length of gestation was collected from linked hospital maternity records. Gestational lengths were calculated using prenatal scan data or last menstrual period dates if scan data were not available. Gestational length was used to calculate the pregnancy trimester periods. We defined first (from day 1 after conception—assumed to be 14 days after last menstrual period to day 92), second (from day 93 to day 184), and third trimester (from day 185 to the end of pregnancy), and the full pregnancy (from day 1 to the end of pregnancy). Self-reports of home addresses reported by the mothers at the first interview (week 26–28) were used to estimate the exposure at spatial level. Information on changing residence during pregnancy was not taken into account.

*Covariates.* Self-reported maternal ethnicity was used to define offspring ethnicity and included in all adjusted models for the full population analyses; it was also used to define the subgroups of stratified analyses. Adjustment variables and coding were selected *a priori* (Anand et al. 2013; Pedersen et al. 2013; Slama et al. 2008): maternal age (years), maternal height (centimeters), maternal pregnancy weight (kilograms) at first gynecological visit, parity (0, 1, ≥ 2), maternal active smoking during pregnancy (yes/no), socioeconomic position based on maternal education (none–primary, A-level equivalent, higher than A-level, other–unknown), and housing tenure [owns or mortgage, rents privately, rents other (council, housing association, other)], sex, gestational age (completed weeks and days, and its square), season of conception [warm season (21 March to 21 September), cold season (22 September to 20 March)]. Information on all covariables were obtained from medical records or from maternal questionnaire (Wright et al. 2013). To evaluate the direct effect of exposure to air pollution on adiposity, models for triceps and subscapular skinfolds were further adjusted for the results of a 2-hr postload plasma glucose test (Fleisch et al. 2014) measured in maternal peripheral blood around 26–28 weeks of gestation using an oral glucose tolerance test (millimoles per liter), which was assayed immediately after sampling at the biochemistry department of Bradford Royal Infirmary, applying the glucose oxidase method of Siemen’s Advia 2400.

*Statistical analysis.* We used linear regression models to evaluate the associations between exposure to air pollution (as a continuous exposure) and birth weight, head circumference at birth, and triceps and subscapular skinfolds thicknesses at birth (as outcomes one at a time). We reported the associations for each 10-μg/m^3^ increase in PM_10_, 5-μg/m^3^ increase in PM_2.5_, 1-10^–5^/m increase in PM_2.5_ absorbance, 10-μg/m^3^ increase in NO_2_, and 20-μg/m^3^ increase in NO_x_. We evaluated the potential effect modification by maternal ethnicity (white British vs. Pakistani) with the inclusion of an interaction term between air pollutants and ethnicity in the models and with model stratification. We used separate regression models to estimate associations with mean exposures during different time windows of pregnancy (full pregnancy and first, second, and third trimester). The covariates adjusted for in these models were listed above. We further adjusted birth weight models for subscapular skinfolds thickness to test whether subscapular skinfolds thickness acted as a mediator on these associations. We generated standardized *z*-scores specific for sex and gestational age (in completed weeks, from 26 to 42) for the full cohort for all the outcomes. We chose to follow the complete case analysis approach.

Sensitivity analyses entailed *a*) exclusion of preterm births (< 37 completed weeks of gestation), *n* = 481 (5%); *b*) exclusion of women who had changed their residence address during pregnancy (because residential mobility could result in exposure misclassification, *n* = 564 (6%); *c*) restriction of birth weight and head circumference analyses to children with triceps skinfold data (to test for selection bias), *n* = 6,188 (68%); *d*) stratification by maternal working status (because nonworkers could spend more time at home and differ in many other unmeasured factors which could cause potential confounding), unemployed, *n* = 4,436 (58%) vs. employed *n* = 3,201 (42%); *e*) stratification by pregnancy maternal smoking status [smokers *n* = 1,645 (19%) vs. nonsmokers *n* = 7,005 (81%)]; and *f*) the exclusion of season of conception from the fully adjusted models (because season of conception can be associated with both exposure and outcome, thus modify their association). We performed all the statistical analyses using Stata/S.E. version 12.1 (StataCorp) and defined statistical significance as an alpha level of 5% (two tails).

## Results

*Study population.* Air pollution exposures and birth weight were available for 4,189 (46%) white British and 4,878 (54%) Pakistani-origin mother–child pairs. White British mothers were younger, were more likely to have a BMI > 25 kg/m^2^ (data not shown), be nulliparous (48% vs. 32%), and have a lower educational level (Table 1) compared with mothers of Pakistani origin. The latter were predominantly nonsmokers, were less likely to be employed, and were less likely to have changed residence during the index pregnancy. The distribution of missing values was not statistically different between the two ethnic groups.

**Table 1:** Population characteristics of the full population and by maternal ethnicity [*n* (%)].

Characteristics	Full population (*n* = 9,067)	White British (*n* = 4,189)	Pakistani origin (*n* = 4,878)	*p*-Value^*a*^
Maternal age (years)				< 0.001
< 20	524 (6)	432 (10)	92 (2)
≥ 20 to < 30	5,275 (58)	2,322 (56)	2,953 (61)
≥ 30 to < 40	3,025 (33)	1,317 (32)	1,708 (35)
≥ 40	225 (3)	109 (3)	116 (2)
Missing	18 (0)	9 (0)	9 (0)
Parity				< 0.001
0 children	3,455 (40)	1,955 (48)	1,500 (32)
1 child	2,511 (29)	1,268 (31)	1,243 (27)
≥ 2 children	2,756 (32)	828 (20)	1,928 (41)
Missing	345 (4)	138 (3)	207 (4)
Maternal education				< 0.001
None/primary	5,066 (56)	2,280 (55)	2,786 (57)
A-level equivalent	1,322 (15)	711 (17)	611 (13)
Higher than A-level	2,048 (23)	793 (19)	1,255 (26)
Other/unknown	607 (7)	399 (10)	208 (4)
Missing	24 (0.3)	6 (0.1)	18 (0.4)
Season of conception^*b*^				0.36
Cold season	4,781 (53)	2,187 (52)	2,594 (53)
Warm season	4,286 (47)	2,002 (48)	2,284 (47)
Sex				0.52
Male	4,664 (51)	2,170 (52)	2,494 (51)
Female	4,403 (49)	2,019 (48)	2,384 (49)
Smoking during pregnancy^*c*^				< 0.001
No	7,005 (81)	2,390 (62)	4,615 (96)
Yes	1,645 (19)	1,462 (38)	183 (4)
Missing	417 (5)	337 (8)	80 (2)
House tenure				< 0.001
Owns or mortage	5,620 (62)	2,197 (53)	3,423 (70)
Rents privately	1,543 (17)	1,050 (25)	493 (10)
Rents other	1,884 (21)	935 (22)	949 (20)
Missing	20 (0.0)	7 (0.0)	13 (0.0)
Type of delivery^*c*^				0.11
At term	8,586 (95)	3,950 (94)	4,636 (95)
Preterm^*d*^	481 (5)	239 (6)	242 (5)
Moved during pregnancy^*c*^				< 0.001
No	8,521 (94)	3,869 (92)	4,652 (95)
Yes	546 (6)	320 (8)	226 (5)
Employment^*c*^				< 0.001
Currently employed	3,201 (42)	2,230 (63)	971 (24)
Never/previously employed	4,436 (58)	1,301 (37)	3,135 (76)
Missing	1,430 (16)	658 (16)	772 (16)
^***a***^Chi-square test for equal distribution of categorical variables; missing values were excluded. ^***b***^Cold season: 22 September to 20 March; warm season: 21 March to 21 September. ^***c***^Used as confounder and/or in a sensitivity analysis. ^***d***^Preterm, gestational age < 37 completed weeks.				

Pakistani-origin participants were exposed to higher mean concentrations of air pollution compared with white British (*p* < 0.01) (see Supplemental Material, Table S1). Pearson correlations between trimester-specific exposures were moderate to low (*r* = –0.06 to 0.57; see Supplemental Material, Table S2). The potential collinearity between PM and NO_x_ or NO_2_ full-pregnancy mean exposures, according to Pearson correlations, was moderate to low (*r* = 0.23 to 0.64; see Supplemental Material, Table S3).

For the full population, the correlation between birth weight and head circumference was high (*r* = 0.73) and did not differ between the two ethnic groups (data not shown). The correlation between birth weight and skinfold thickness was modest amongst both the white British (*r* = 0.50) and Pakistani-origin children (*r* = 0.55). The correlation between head circumference and skinfold thickness was low in both groups (*r* = 0.35 in white British and *r* = 0.30 in children of Pakistani origin). All the correlations above were statistically significant. Girls of both ethnicities had on average lower birth weights (≈ 180 g), smaller head circumferences (≈ 0.5 cm), and higher skinfold thicknesses (≈ 0.2 mm) than boys; these sex differences were greater among Pakistani-origin children than white British children (data not shown).

*Associations between air pollution and newborn size.* In the full population we generally observed inverse associations between air pollution and birth weight in the adjusted analyses (see Table 2 for associations with exposures during the full pregnancy and third trimester; see also Supplemental Material, Table S4 for associations with exposures during the first and second trimesters), but none of the associations were statistically significant. We observed a statistically significant decrease in head circumference at birth for exposures to PM_10_ and PM_2.5_ during the entire pregnancy and all trimesters and for NO_x_ during the second trimester. We observed that PM_10_ and PM_2.5_ exposures were positively associated with triceps and subscapular skinfold thicknesses, with most associations being statistically significant. In contrast, most associations were null or inverse for exposure to PM_2.5_ absorbance, NO_2_, and NO_x_ during the entire pregnancy and during each trimester.

**Table 2:** Adjusted*^a^* model coefficients (95% CI) for air pollution exposure*^b^* with birth weight (g), head circumference (cm), and triceps (mm) and subscapular skinfold thickness (mm).

Outcome/ air pollutant	Pregnancy period	Full population	*p*-Value of interaction^*c*^	White British	Pakistani origin
Birth weight		*n *= 7,969		*n *= 3,601	*n *= 4,368
PM_10_	Full pregnancy	–9 (–41, 23)	0.16	–36 (–83, 10)	23 (–21, 66)
	3rd trimester	–13 (–42, 16)	0.15	–39 (–82, 4)	13 (–27, 52)
PM_2.5_	Full pregnancy	–11 (–33, 1)	0.03	–45 (–81, –9)	15 (–14, 43)
	3rd trimester	–12 (–33, 8)	0.03	–43 (–76, –10)	9 (–17, 35)
PM_2.5_ absorbance	Full pregnancy	–5 (–53, 44)	0.01	–64 (–135, 7)	49 (–17, 115)
	3rd trimester	–8 (–47, 31)	0.12	–45 (–104, 14)	22 (–31, 75)
NO_2_	Full pregnancy	9 (–15, 34)	0.05	–15 (–52, 23)	24 (–8, 56)
	3rd trimester	4 (–13, 22)	0.13	–13 (–40, 14)	15 (–8, 38)
NO_x_	Full pregnancy	–4 (–27, 18)	0.06	–27 (–62, 8)	12 (–18, 41)
	3rd trimester	–2 (–20, 16)	0.14	–19 (–46, 10)	10 (–14, 33)
Head circumference		*n *= 7,330		*n *= 3,301	*n *= 4,029
PM_10_	Full pregnancy	–0.28 (–0.38, –0.17)	0.41	–0.32 (–0.47, –0.17)	–0.23 (–0.37, –0.08)
	3rd trimester	–0.22 (–0.32, –0.13)	0.01	–0.32 (–0.46, –0.18)	–0.14 (–0.27, –0.01)
PM_2.5_	Full pregnancy	–0.19 (–0.27, –0.12)	0.04	–0.29 (–0.41, –0.17)	–0.13 (–0.22, –0.03)
	3rd trimester	–0.16 (–0.23, –0.09)	< 0.001	–0.28 (–0.39, –0.17)	–0.08 (–0.17, 0.01)
PM_2.5_ absorbance	Full pregnancy	–0.12 (–0.28, 0.03)	0.09	–0.23 (–0.46, 0.00)	–0.04 (–0.25, 0.18)
	3rd trimester	–0.06 (–0.18, 0.07)	0.01	–0.18 (–0.37, 0.01)	0.03 (–0.14, 0.20)
NO_2_	Full pregnancy	–0.01 (–0.09, 0.07)	0.74	0.00 (–0.12, 0.12)	–0.01 (–0.12, 0.09)
	3rd trimester	0.05 (–0.01, 0.11)	0.16	0.03 (–0.06, 0.12)	0.06 (–0.01, 0.14)
NO_x_	Full pregnancy	–0.08 (–0.15, 0.00)	0.99	–0.05 (–0.17, 0.06)	–0.08 (–0.18, 0.01)
	3rd trimester	–0.01 (–0.07, 0.05)	0.38	–0.01 (–0.10, 0.09)	–0.01 (–0.09, 0.07)
Triceps skinfolds		*n *= 5,316		*n *= 2,254	*n *= 3,062
PM_10_	Full pregnancy	0.15 (0.04, 0.26)	0.23	0.06 (–0.10, 0.23)	0.23 (0.09, 0.37)
	3rd trimester	0.13 (0.03, 0.23)	0.18	0.03 (–0.13, 0.19)	0.21 (0.08, 0.34)
PM_2.5_	Full pregnancy	0.11 (0.03, 0.18)	0.05	0.00 (–0.13, 0.13)	0.18 (0.09, 0.27)
	3rd trimester	0.10 (0.03, 0.17)	0.06	–0.02 (–0.14, 0.10)	0.17 (0.08, 0.25)
PM_2.5_ absorbance	Full pregnancy	–0.06 (–0.22, 0.09)	0.32	–0.15 (–0.39, 0.10)	0.00 (–0.20, 0.20)
	3rd trimester	0.02 (–0.11, 0.14)	0.34	–0.08 (–0.28, 0.12)	0.08 (–0.08, 0.24)
NO_2_	Full pregnancy	–0.19 (–0.26, –0.11)	0.28	–0.23 (–0.36, –0.11)	–0.17 (–0.26, –0.07)
	3rd trimester	–0.05 (–0.11, 0.00)	0.38	–0.09 (–0.18, 0.00)	–0.03 (–0.10, 0.04)
NO_x_	Full pregnancy	–0.10 (–0.17, –0.02)	0.03	–0.20 (–0.32, –0.08)	–0.03 (–0.12, 0.05)
	3rd trimester	–0.02 (–0.08, 0.03)	0.11	–0.10 (–0.19, 0.00)	0.02 (–0.05, 0.09)
Subscapular skinfolds		*n *= 5,302		*n *= 2,247	*n *= 3,055
PM_10_	Full pregnancy	0.20 (0.09, 0.31)	0.20	0.12 (–0.05, 0.29)	0.28 (0.13, 0.42)
	3rd trimester	0.21 (0.10, 0.31)	0.25	0.13 (–0.02, 0.29)	0.27 (0.13, 0.40)
PM_2.5_	Full pregnancy	0.15 (0.07, 0.22)	0.05	0.04 (–0.09, 0.17)	0.21 (0.12, 0.31)
	3rd trimester	0.15 (0.08, 0.22)	0.11	0.06 (–0.06, 0.18)	0.21 (0.12, 0.29)
PM_2.5_ absorbance	Full pregnancy	0.08 (–0.08, 0.24)	0.42	0.03 (–0.21, 0.27)	0.11 (–0.09, 0.32)
	3rd trimester	0.04 (–0.08, 0.17)	0.31	–0.05 (–0.24, 0.15)	0.10 (–0.06, 0.26)
NO_2_	Full pregnancy	0.00 (–0.08, 0.08)	0.71	0.00 (–0.13, 0.12)	–0.01 (–0.11, 0.09)
	3rd trimester	–0.02 (–0.08, 0.03)	0.67	–0.04 (–0.12, 0.05)	–0.02 (–0.09, 0.05)
NO_x_	Full pregnancy	0.01 (–0.06, 0.09)	0.35	–0.02 (–0.14, 0.10)	0.03 (–0.06, 0.13)
	3rd trimester	–0.01 (–0.06, 0.05)	0.41	–0.04 (–0.13, 0.06)	0.02 (–0.06, 0.09)
^***a***^Birth weight and head circumference models were adjusted for gestational age (complete weeks and days) and (complete weeks and days)^2^, maternal age (years), parity (0, 1, ≥ 2), socioeconomic position (maternal education and house tenure), maternal height (cm), maternal weight at examination (kg), maternal active tobacco smoking during pregnancy (yes, no), season of conception (cold, warm), and sex. Triceps and subscapular skinfolds models were further adjusted for maternal 2-hr postload glucose at 26–28 weeks (mmol/L). ^***b***^The following increments were used: 10-μg/m^3^ increase in PM_10_, 5-μg/m^3^ increase in PM_2.5_, 1-10^–5^/m increase in PM_2.5_ absorbance, 10-μg/m^3^ increase in NO_2_, and 20-μg/m^3^ increase in NO_x_. ^***c***^Interaction between the indicated air pollutant (continuous variable) and maternal ethnicity (white/Pakistani origin).					

*Effect modification by maternal ethnicity.* For birth weight analyses we found various statistically significant interactions (*p* ≤ 0.05) between maternal ethnicity and exposures to air pollution (Table 2; see also Supplemental Material, Table S4). The stratified analyses showed inverse association between exposure to air pollution and birth weight in white British infants but not in Pakistani-origin infants, which reached the level of statistical significance for PM_2.5_—a 5-μg/m^3^ increase in exposure during the third trimester—and was associated with a reduction of 43 g [95% confidence interval (CI): –76, –10] in white British infants versus a nonsignificant increment of 9 g (95% CI: –17, 35) in Pakistani-origin infants. For head circumference we also found statistically significant interactions (*p* ≤ 0.05). In the stratified analyses, the point estimates of the inverse associations with PM were almost double in magnitude in white British compared with Pakistani-origin children. For example a 5-μg/m^3^ increase in PM_2.5_ exposure during the third trimester was associated with a reduction of 0.28 cm (95% CI: –0.39, –0.17) in white British infants and a reduction of 0.08 cm (95% CI: –0.17, 0.01) in Pakistani-origin infants.

For triceps and subscapular skinfold thickness, we found statistically significant interactions (*p* ≤ 0.1) between PM_2.5_, NO_x_, and ethnicity. A 5-μg/m^3^ increase in PM_2.5_ during the third trimester was not associated with these outcomes [–0.02 mm (95% CI: –0.14, 0.10) for triceps skinfold thickness and 0.06 mm (95% CI: –0.06, 0.18) for subscapular skinfold thickness] in the white British population, whereas it was associated with an increment of 0.17 mm (95% CI: 0.08, 0.25) in triceps and of 0.21 mm (95% CI: 0.12, 0.29) in subscapular thickness in Pakistani-origin population. On the contrary, a 20-μg/m^3^ increase in exposure to NO_x_ during the full pregnancy was associated with a reduction of 0.20 mm (95% CI: –0.32, –0.08) in triceps skinfolds in white British babies, but not in Pakistani-origin babies (–0.03 mm; 95% CI: –0.12, 0.05).

*Adiposity as a mediator.* We further adjusted the birth weight models for subscapular skinfold thickness (Table 3). In the full population, the association between a 5-μg/m^3^ increase in PM_2.5_ during the third trimester and birth weight became statistically significant (–28 g; 95% CI: –51, –6) (Table 3, model 2), whereas it was not in the main model (1 g; 95% CI: –25, 27) (Table 3, model 1). Furthermore the differences between the two ethnic groups attenuated (β_whiteBritish_ = –50 g; 95% CI: –88, –12 vs. β_Pakistani_ = –14 g; 95% CI: –42, 14; *p*_interaction_ = 0.17) compared with the difference observed in the main analyses, and all the interactions became non-statistically significant (Table 3).

**Table 3:** Restriction to those with measured triceps thickness (*n* = 6,188) exposure to the 3rd trimester, model 1 [adjusted*^a^* models for air pollution exposure*^b^* and birth weight (g) vs. model 2 (further adjusted for subscapular skinfolds thickness] [coefficients (95% CI)].

Air pollutant	Model^*a*^	Full population (*n* = 5,482)	*p*-Value of interaction^*c*^	White British (*n* = 2,337)	Pakistani origin (*n* = 3,145)
PM_10_	Model 1	6 (–31, 44)	0.10	–34 (–92, 23)	41 (–8, 90)
	Model 2	–32 (–64, 0)	0.21	–57 (–107, –7)	–10 (–52, 31)
PM_2.5_	Model 1	1 (–25, 27)	0.05	–38 (–82, 5)	27 (–6, 60)
	Model 2	–28 (–51, –6)	0.17	–50 (–88, –12)	–14 (–42, 14)
PM_2.5_ absorbance	Model 1	10 (–36, 57)	0.14	–39 (–112, 34)	44 (–16, 105)
	Model 2	–2 (–42, 38)	0.34	–29 (–92, 35)	16 (–35, 68)
NO_2_	Model 1	7 (–13, 27)	0.25	–11 (–43, 22)	17 (–9, 43)
	Model 2	9 (–8, 27)	0.32	–5 (–33, 23)	17 (–5, 40)
NO_x_	Model 1	–1 (–22, 21)	0.22	–20 (–55, 14)	12 (–15, 40)
	Model 2	–2 (–20, 16)	0.39	–14 (–44, 16)	6 (–18, 29)
^***a***^Models were adjusted for gestational age (complete weeks and days) and (complete weeks and days)^2^, maternal age (years), parity (0, 1, ≥ 2), socioeconomic position (maternal education and house tenure), maternal height (cm), maternal weight at examination (kg), maternal active tobacco smoking during pregnancy (yes, no), season of conception (cold, warm), and sex. Full population models were adjusted also for ethnicity (white British, Pakistani origin); model 2 was further adjusted for subscapular skinfold thickness. ^***b***^Effect estimates correspond to 10-μg/m^3^ increase in PM_10_, 5-μg/m^3^ increase in PM_2.5_, 1-10^–5^/m increase in PM_2.5_ absorbance, 10-μg/m^3^ increase in NO_2_, and 20-μg/m^3^ increase in NO_x_. ^***c***^Interaction between the indicated air pollutant and maternal ethnicity.					

**Figure 1:**
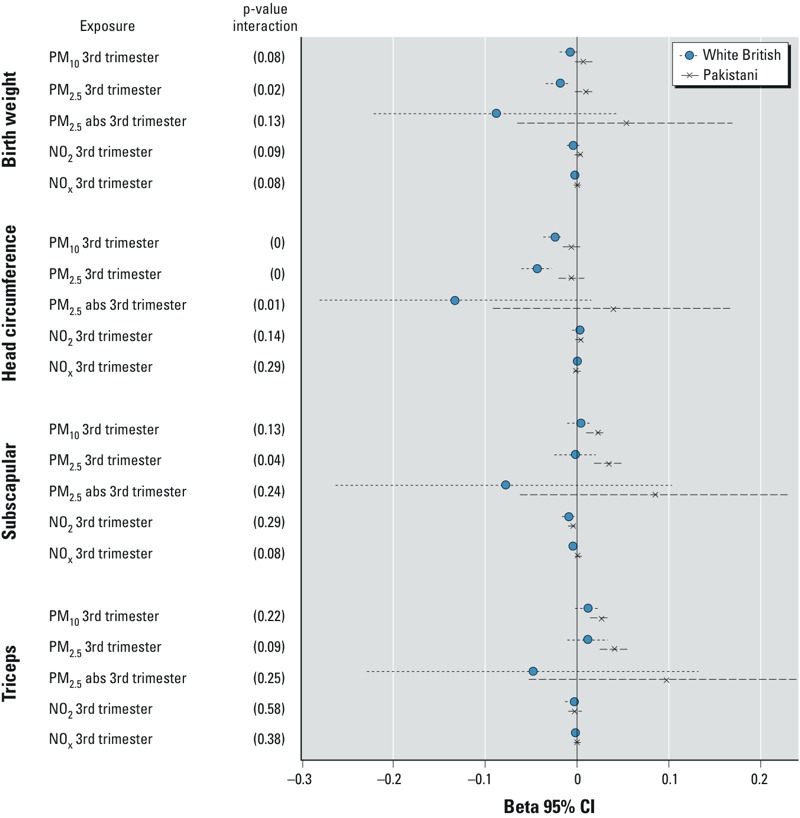
*z*-Scores for adjusted*^a^* models coefficients (95% CI)*^b^* and *p*-value of interaction*^c^* for air pollution exposure and birth weight, head circumference, triceps skinfold thickness, and subscapular skinfold thickness, stratified by ethnicity. ***^a^***Birth weight and head circumference models adjusted for maternal age (years), parity (0, 1, ≥ 2), socioeconomic position (maternal education and house tenure), maternal height (cm), maternal weight at examination (kg), maternal active tobacco smoking during pregnancy (yes, no), and season of conception (cold, warm). Triceps and subscapular skinfolds models were further adjusted for maternal 2-hr postload glucose at 26–28 weeks. ***^b^***Effect estimates correspond to 10-μg/m^3^ increase in PM_10_, 5-μg/m^3^ increase in PM_2.5_, 1-10^–5^/m increase in PM_2.5_ absorbance (abs), 10-μg/m^3^ increase in NO_2_, and 20-μg/m^3^ increase in NO_x_. ***^c^***Interaction between the indicated air pollutant and maternal ethnicity.

*Sensitivity analyses.* The results were similar when we analysed sex- and age-standardized *z*-score values to those reported (Figure 1). The reported results were also robust to restriction to term birth; nonmovers during pregnancy (see Supplemental Material, Tables S5 and S6), and to subjects with available triceps skinfold measurements (Table 3). Results from analyses by maternal employment status showed that newborns of employed mothers had somewhat stronger decrease in birth weight and smaller head circumference compared with newborns of unemployed mothers, and do not show a clear pattern for skinfold thicknesses (see Supplemental Material, Table S7). There was little evidence of an influence of smoking on associations between the exposure to air pollution and active maternal smoking (see Supplemental Material, Table S8). After removing season of conception from the full adjusted models, the association between air pollution and birth weight in the full population was strengthened for all the exposures, and reached the statistical significance level for exposure to PM_2.5_ during the third trimester (–20 g; 95% CI: –38, –2) (results not shown), suggesting that season of conception was a mediator in the association of exposure to air pollution and birth weight in our study.

## Discussion

In the present study, exposure to PM was associated with child size at birth: We found a reduction in mean birth weight in white British infants, but not in those of Pakistani origin. Ethnic differences were also found in the associations between air pollution and head circumference and triceps and subscapular skinfold thickness. Despite the association between exposure to PM and subscapular skinfold being clinically negligible, its potential mechanistic role was strengthened by the assessment of the potential mediating role of this body fat indicator in the associations between prenatal exposure to airborne PM and birth weight in Pakistani-origin infants (Table 3). PM was positively associated with birth weight among Pakistani-origin babies before adjustment, whereas associations after adjustment for skinfold thickness were essentially null, suggesting that the main effect on birth weight was through this fat indicator among this ethnic group.

Birth weight is recognized and used in epidemiological studies as indicator of intrauterine growth. Birth weight reflects both the skeletal mass and the mass growth composed by the lean mass and the adipose tissues. Skeletal growth can also be measured at birth as the length of the newborn or estimated through ultrasound scan measures as femoral length (Estarlich et al. 2011), which have been used in environmental epidemiology studies (Dadvand et al. 2013; Pedersen et al. 2013; Slama et al. 2008; Stieb et al. 2012). Skinfold thickness can be used to measure the lean and adipose tissue of the newborn and children as they grow. Head circumference at birth has been suggested to indicate brain growth, and child head circumference has been associated with intelligence and cognitive function (Gale et al. 2004), but this is not yet well established.

We found a non-statistically significant association between exposure to PM and mean birth weight [a 5-μg/m^3^ increase in the exposure to PM_2.5_ during the full pregnancy was associated with a reduction of 11 g (95% CI: –33, 1)]; however, the point estimates are consistent with those found in a larger study by Pedersen et al. (2013) (10 g; 95% CI: –19, 0), a meta-analysis by Stieb et al. (2012) (12 g; 95% CI: –23, –1) and in a recent study by Basu et al. (2014) (–5 g; 95% CI: –6, –3). The associations in our study between PM and head circumference were much stronger than those observed by Pedersen et al. (2013): In our study, a 5-μg/m^3^ increase in the exposure to PM_2.5_ during the full pregnancy was associated with a reduction of 19 cm (95% CI: –0.27, –0.12) in head circumference, whereas Pedersen et al. (2013) reported a mean reduction of 0.08 cm (95% CI: –0.12, –0.03). However, in the present study the reduction in head circumference was evident only for PM, not for NO_2_ and NO_x_, which was not the case in the larger ESCAPE sample in which associations for head circumference were evident for all pollutants (Pedersen et al. 2013). These differences could be attributed to differences in chemical constituents, sources, and levels of PM between the study areas, the different composition of the study population, and/or perhaps residual differences in study populations (Basu et al. 2014; Stieb et al. 2012; Woodruff et al. 2009). Ambient PM in the city of Bradford may have a potentially higher toxicity because of the industrial sites nearby and because of the high proportion of heavy vehicles that cross the city for the transportation of goods (Jones 2009).

The findings of the present study provide no or little evidence of a reduction in the mean birth weight associated with prenatal exposures to NO_2_ and NO_x_, similar to the findings reported by Pedersen et al. (2013) and by Stieb et al. (2012).

*Ambient air pollution and adiposity.* To our knowledge, this study is the first to examine the associations between prenatal exposure to air pollution and adiposity in newborns. Our results suggest that exposure to higher levels of PM are associated with greater adiposity at birth. An experimental study reported increased adiposity in mice offspring after gestational exposure to PM_2.5_ (Bolton et al. 2012). The increased adiposity might be explained by an exacerbation of the adipose tissue inflammation and insulin resistance, as suggested in another mice experiment (Sun et al. 2009). Maternal smoking during pregnancy, in humans, has been associated with reduced birth weight, head circumference, and length at birth, but was not found to be associated with the total fat mass of the newborns at birth (Bernstein et al. 2000; D’Souza et al. 1981; Luciano et al. 1998). Maternal smoking during pregnancy has also been associated with increased risk of obesity during childhood (Durmus et al. 2014).

The literature presented above and the results of the present study highlight the importance of examination of supplemental measures such as skinfold thickness, in addition to the more classical measurement of birth weight in studies aiming to characterize the intrauterine growth in terms of fat mass, lean mass, skeletal growth, and head growth. Further epidemiological evidence on the association between prenatal exposure to ambient air pollution and more specific outcomes of *in utero* growth such as skinfold thickness would offer more insights on the possible biological mechanisms underlying the observed associations.

Skinfold measurements have previously been used to monitor adiposity and adiposity growth in children (Krishnaveni et al. 2005; Misra and Khurana 2009; West et al. 2013; Yajnik et al. 2002, 2003). Skinfold measurements in newborns have been associated with the risks of obesity and cardiovascular disease later in life (Patel and Abate 2013; Snijder et al. 2006; Yajnik et al. 2002). The use of skinfolds to measure adiposity is relatively noninvasive, simple, cheap, and reliable compared with other, more sophisticated techniques such as dual-energy X-ray absorptiometry or densiometry (Wells and Fewtrell 2006).

*Effect modification by maternal ethnicity.* Our results suggest that newborn participants in BiB of white British origin have a slightly higher susceptibility to air pollution than those of Pakistani origin for some outcomes. In particular, in white British participants, prenatal exposure to PM was associated with a decrease in the mean birth weight, but not in the mean fat mass (Table 2), whereas in the Pakistani population, exposure to PM was associated not with a decrease in the mean birth weight, but instead with an increase in the mean fat mass. In models that were further adjusted for fat mass, exposure to PM was associated with a reduction in the mean birth weight in the Pakistani population, as it was in the white British population (Table 3). Previous evidence on effect modification by ethnicity is inconsistent between studies and pollutants. Negative associations between ambient air pollution and birth weight have been reported to be stronger in non-Hispanic black compared with non-Hispanic white and Hispanic populations in some previous studies (Basu et al. 2014; Bell et al. 2010; Darrow et al. 2011), whereas other studies have reported weaker associations among Asians, African Americans, and Hispanics, compared with non-Hispanic whites (Basu et al. 2014; Geer et al. 2012). South Asian populations are underrepresented in the air pollution–birth outcome studies, so it is hard to generalize results under the label of “ethnic differences.” However, the inclusion of anthropometric characteristic of newborns, such as direct indicators of fat mass, can help explain such differences.

The adipose tissue overflow hypothesis (Bhopal 2002; Sniderman et al. 2007) describes how the fat compartments and their distribution vary between whites and South Asians. South Asians seem to be more prone to central adiposity, which has been associated with higher risk of cardiometabolic syndrome (Bhopal 2002; Misra and Khurana 2009; Patel and Abate 2013).

Ambient air pollution exposure during pregnancy has been associated with altered placental mitochondrial function in humans (Janssen et al. 2012), which in turn has been proposed to be associated with the susceptibility to adiposity (Bhopal and Rafnsson 2009). In particular, mitochondrial efficiency has been suggested to contribute to the factors that could explain why South Asian populations are more prone to store fat in deep visceral adipose tissue depots compared with white populations (Bhopal and Rafnsson 2009).

*Strengths and limitations.* A main strength of the study is the use of a fine-scale exposure assessment based on temporal adjustment of LUR in a multi-ethnic cohort with information on a range of potential confounders and additional measures of newborn size.

This exposure assessment approach relies on the assumption that the spatial distribution of pollutants within the cities and their determinants remain constant over the study period, which has been confirmed before by comparing two models developed in the same city, using the same methodology at 10 years apart (Eeftens et al. 2011).

We cannot rule out the potential exposure misclassification that may have arisen from the lack of assessment of nonresidential exposures, from the lack of assessment of residential mobility during pregnancy, and from assumptions needed for the temporal adjustment of the LUR models. As a surrogate for nonresidential exposure, we used data on working status (15% missing) (see Supplemental Material Table S7). However, the results from this analysis should be interpreted with caution because of the large proportion of missing values in this covariate and because in our study population the employed mothers were predominantly white British; furthermore, we cannot exclude exposure misclassification because employed status can be associated with other factors such as maternal or familial diseases and poverty. In our study population, 6% of women had changed residence during pregnancy, and excluding them from the analyses did not substantially change the results (see Supplemental Material, Tables S6).

The differences in effect estimates by ethnicity are complex and could be further related to differences in air pollution composition or housing quality or differences in other risk factors for the outcomes studied such as socioeconomic disadvantages, younger age, poor nutritional status, poor prenatal health care, co-exposure to other pollutants, stress, and instability in residential settings (O’Neill et al. 2003). However, we did adjust for socioeconomic position of the mother, combining data on maternal education and house tenure. We did not account for maternal diet in our analyses. In a subsample of our cohort, a higher amount of fresh fruit and sugar-sweetened beverages was found in the homes of Pakistani origin participants compared with white British (Bryant et al. 2015), but this might reflect the differences between the two ethnic groups in family size and other differences in terms of other dietary patterns, maternal health, maternal occupation, and time–activity patterns, which are still to be assessed in future studies.

## Conclusion

Our findings from a large multi-ethnic British birth cohort suggest that *in utero* exposure to PM may influence the size of newborns in a differential manner between white British and Pakistani-origin babies. The associations between ambient airborne PM and skinfold measures in newborns deserve further study and require replication in different settings and confirmation that associations persist as the cohort increases in age.

Further assessment of airborne toxicants such as polycyclic aromatic hydrocarbons and the elemental composition of PM, as well as genetic and lifestyle (e.g., diet) factors could help further our understanding of mechanisms that may explain the differences in associations between birth size and adiposity between ethnic groups observed in our study.

## Supplemental Material

(381 KB) PDF
