# Supplementary material for: Ambient Air Pollution and Newborn Size and Adiposity at Birth: Differences by Maternal Ethnicity (the Born in Bradford Study Cohort)
Source: Environ Health Perspect. 2015 May 15;123(11):1208–15. doi: 10.1289/ehp.1408675 (PMC4629735; doi:10.1289/ehp.1408675)
Supplement: (381 KB) PDF [file ehp.1408675.s001.acco.pdf]

Environ Health Perspect
doi: 10.1289/ehp.1408675

Note to Readers: *EHP* strives to ensure that all journal content is accessible to all readers. However, some figures and Supplemental Material published in *EHP* articles may not conform to 508 standards due to the complexity of the information being presented. If you need assistance accessing journal content, please contact ehp508@niehs.nih.gov. Our staff will work with you to assess and meet your accessibility needs within 3 working days.

Supplemental Material

Ambient Air Pollution and Newborn Size and Adiposity at Birth: Differences by Maternal Ethnicity (the Born in Bradford Study Cohort)

Anna Schembari, Kees de Hoogh, Marie Pedersen, Payam Dadvand, David Martinez, Gerard Hoek, Emily S. Petherick, John Wright, and Mark J. Nieuwenhuijsen

Table of Contents

Table S1. Characteristics and exposure distributions for the full population and by maternal ethnicity.

Table S2. Pearson correlations between trimester specific exposure and the full pregnancy for each air pollutant.

Tables S3. Pearson correlations between air pollutant exposures for the full pregnancy.

Table S4. Adjusted models coefficients (95% CI) for air pollution exposure during 1st and 2nd trimester of pregnancy with birth weight (g) head circumference (cm), triceps and subscapular skinfolds thickness (mm).

Table S5. Term babies (N=8,586) - Adjusted models coefficients (95% CI) for air pollution exposure and birth weight (g) head circumference (cm), triceps and subscapular skinfolds thickness (mm).

Table S6. Mothers who don't change residence during pregnancy (N=8,521) - Adjusted models coefficients (95% CI) for ambient air pollution exposure and birth weight (g), head circumference (cm), triceps and subscapular skinfolds thickness (mm).

Table S7. Unemployed (n=4,436) vs. employed (n=3,201) mothers - Adjusted models coefficients (95% CI) for air pollution exposure and birth weight (g) head circumference (cm) triceps and subscapular skinfolds thickness (mm).

Table S8. Non-smokers (N=7,005) vs smoker (N=1,645) mothers - Adjusted models coefficients (95% CI) for air pollution exposure and birth weight (g), head circumference (cm), triceps and subscapular skinfolds thickness (mm).

Table S1. Characteristics and exposure distributions for the full population and by maternal ethnicity.

	Full Population		White British		Pakistani origin	
**Characteristics**	**N (%)[a]**	**Mean (p5-p95)**	**N (%)[a]**	**Mean (p5-p95)**	**N (%)[a]**	**Mean (p5-p95)**
Maternal age (years)	9,049 (0)	27.7 (19.0 38.0)	4,180 (0)	27.1 (18.0-38.0)	4,869 (0)	28.2 (21.0-38.0)
Maternal weight (kg)	8,737 (4)	74.4 (53.6-103.1)	4,064 (3)	78.0 (56.2-109.9)	4,673 (4)	71.3 (52.1-96.7)
Maternal height (cm)	8,867 (2)	161.7 (151.7-172.4)	4,123 (2)	164.0 (153.9-174.2)	4,744 (3)	159.7 (150.6-169.0)
2-h postload Glucose (mmol/L)	8,685 (4)	5.7 (3.7-8.2)	3,997 (5)	5.4 (3.6-7.7)	4,688 (4)	5.9 (3.9-8.8)
Gestational age (weeks and days)	9,067 (0)	39.6 (36.7-41.7)	4,189 (0)	39.7 (36.4-41.7)	4,878 (0)	39.5 (37.0-41.6)
Birth weight (g)	9,067 (0)	3,244 (2,360-4,110)	4,189 (0)	3,363 (2,470-4,220)	4,878 (0)	3,142 (2,320-3,960)
Birth head circumference (cm)	8,311 (8)	34.3 (31.9-36.6)	3,830 (9)	34.5 (32.0-37.0)	4,481 (8)	34.1 (31.5-36.3)
Birth Triceps skinfold (mm)	6,188 (32)	5.1 (3.4-7.0)	2,718 (35)	5.2 (3.6-7.2)	3,470 (29)	5.0 (3.4-7)
Birth Subscapular skinfold (mm)	6,169 (32)	4.7 (3.2-6.8)	2,709 (35)	4.8 (3.2-6.8)	3,460 (29)	4.7 (3.0-6.8)
PM_{10} full pregnancy (μg/m^3)	9,067 (0)	18.6 (14.8-23.6)	4,189 (0)	18.5 (14.6-23.6)	4,878 (0)	18.7 (14.9-23.7)
PM_{10} 3rd trimester (μg/m^3)	9,063 (0)	18.2 (12.9-23.9)	4,188 (0)	18.1 (12.8-23.8)	4,875 (0)	18.3 (13.0-24.0)
$PM_{2.5}$ full pregnancy (μg/m^3)	9,067 (0)	12.7 (9.7-16.7)	4,189 (0)	12.0 (9.3-15.3)	4,878 (0)	13.3 (10.1-17.3)
$PM_{2.5}$ 3rd trimester (μg/m^3)	9,063 (0)	12.4 (8.6-16.6)	4,188 (0)	11.8 (8.3-15.4)	4,875 (0)	13.0 (9.0-17.1)
$PM_{2.5}$ abs. full pregnancy (10^{-5}/m)	9,067 (0)	1.27 (1.03-1.58)	4,189 (0)	1.22 (1.00-1.55)	4,878 (0)	1.31 (1.08-1.60)
$PM_{2.5}$ abs. 3rd trimester (μg/m^3)	9,063 (0)	1.27 (0.86-1.76)	4,188 (0)	1.23 (0.82-1.71)	4,875 (0)	1.31 (0.89-1.78)
NO_2 full pregnancy (μg/m^3)	9,065 (0)	21.4 (15.6-28.2)	4,187 (0)	20.2 (15.0-26.3)	4,878 (0)	22.4 (16.5-29.1)
NO_2 3rd trimester (μg/m^3)	9,061 (0)	21.1 (12.7-32.7)	4,186 (0)	20.0 (12.3-31.0)	4,875 (0)	22.1 (13.5-33.8)
NO_x full pregnancy (μg/m^3)	9,065 (0)	36.0 (23.9-50.5)	4,187 (0)	33.4 (22.5-46.7)	4,878 (0)	38.1 (25.6-51.9)
NO_x 3rd trimester (μg/m^3)	9,061 (0)	35.4 (20.2-56.8)	4,186 (0)	33.0 (19.1-53.2)	4,875 (0)	37.4 (21.6-58.7)

[a]Percentage of missing within each group.

All characteristics differed statistically significantly between British and the Pakistani origin according to ANOVA test for difference in mean between the two groups, significant at α level (α =0.05)

Table S2. Pearson correlations[a] between trimester specific exposure and the full pregnancy for each air pollutant.

		Pregnancy period		
**Air pollutant**	**Pregnancy period**	**Full pregnancy**	**3rd trimester**	**2nd trimester**
PM_{10}	3rd trimester	0.71		
	2nd trimester	0.85	0.44	
	1st trimester	0.77	0.28	0.51
$PM_{2.5}$	3rd trimester	0.75		
	2nd trimester	0.86	0.50	
	1st trimester	0.80	0.36	0.57
$PM_{2.5}$ absorbance	3rd trimester	0.57		
	2nd trimester	0.85	0.31	
	1st trimester	0.55	-0.24	0.36
NO_2	3rd trimester	0.47		
	2nd trimester	0.80	0.19	
	1st trimester	0.50	-0.34	0.23
NO_x	3rd trimester	0.62		
	2nd trimester	0.84	0.37	
	1st trimester	0.64	-0.06	0.40

[a]All the correlations were statistically significant at α level<0.01, number of subjects are presented in Table S1.

Tables S3. Pearson correlations[a] between air pollutant exposures for the full pregnancy.

**Air pollutant**	**PM_{10}**	**$PM_{2.5}$**	**$PM_{2.5}$ abs.**	**NO_2**
$PM_{2.5}$	0.80			
$PM_{2.5}$ absorbance	0.48	0.44		
NO_2	0.23	0.36	0.63	
NO_x	0.45	0.53	0.64	0.83

[a]All the correlations were statistically significant at α level<0.01, number of subjects are presented in Table S1 .

doi: 10.1289/ehp.1408675

Table S4. Adjusted[a] models coefficients (95% CI) for air pollution exposure[b] during 1st and 2nd trimester of pregnancy with birth weight (g) head circumference (cm), triceps and subscapular skinfolds thickness (mm).

Outcome	Air pollutant	Pregnancy period	Full population	p-value of int.[c]	White British	Pakistani origin
Birth Weight			N=7,969		N=3,601	N=4,368
	PM_{10}	2nd trimester	-9 (-32, 15)	0.32	-23 (-57, 12)	10 (-22, 42)
		1st trimester	4 (-23, 31)	0.40	-15 (-55, 25)	24 (-13, 61)
	$PM_{2.5}$	2nd trimester	-8 (-25, 8)	0.11	-26 (-53, 1)	7.0 (-15, 29)
		1st trimester	-1 (-21, 18)	0.13	-26 (-57, 5)	17 (-8, 41)
	$PM_{2.5}$ absorbance	2nd trimester	-11 (-43, 21)	0.02	-48 (-96, 0)	22 (-21, 64)
		1st trimester	14 (-27, 55)	0.31	-18 (-79, 43)	39 (-16, 95)
	NO_2	2nd trimester	1 (-13, 15)	0.17	-9 (-31, 14)	7 (-11, 26)
		1st trimester	9 (-9, 27)	0.65	2 (-27, 31)	11 (-13, 35)
	NO_x	2nd trimester	-4 (-19, 12)	0.14	-16 (-40, 9)	5 (-15, 25)
		1st trimester	0 (-19, 18)	0.43	-12 (-41, 18)	6 (-18, 30)
Head circumference			N=7,330		N=3,301	N=4,029
	PM_{10}	2nd trimester	-0.21 (-0.28, -0.13)	0.93	-0.20 (-0.31, -0.09)	-0.20 (-0.30, -0.09)
		1st trimester	-0.15 (-0.24, -0.06)	0.95	-0.19 (-0.32, -0.05)	-0.11 (-0.24, 0.01)
	$PM_{2.5}$	2nd trimester	-0.15 (-0.20, -0.09)	0.40	-0.18 (-0.27, -0.09)	-0.12 (-0.19, -0.05)
		1st trimester	-0.11 (-0.17, -0.05)	0.42	-0.18 (-0.28, -0.08)	-0.06 (-0.14, 0.02)
	$PM_{2.5}$ absorbance	2nd trimester	-0.10 (-0.21, 0.00)	0.18	-0.14 (-0.30, 0.02)	-0.07 (-0.21, 0.07)
		1st trimester	-0.05 (-0.18, 0.08)	0.71	-0.10 (-0.30, 0.09)	0.00 (-0.18, 0.18)
	NO_2	2nd trimester	-0.03 (-0.08, 0.02)	0.49	-0.03 (-0.10, 0.04)	-0.03 (-0.09, 0.03)
		1st trimester	-0.02 (-0.08, 0.04)	0.15	0.01 (-0.08, 0.10)	-0.04 (-0.11, 0.04)
	NO_x	2nd trimester	-0.06 (-0.11, -0.01)	0.62	-0.05 (-0.13, 0.02)	-0.06 (-0.13, 0.00)
		1st trimester	-0.05 (-0.11, 0.01)	0.19	-0.02 (-0.12, 0.07)	-0.07 (-0.15, 0.01)

Outcome	Air pollutant	Pregnancy period	Full population	p-value of int.[c]	White British	Pakistani origin
Triceps skinfolds			N=5,316		N=2,254	N=3,062
	PM_{10}	2nd trimester	0.01 (-0.07, 0.10)	0.45	-0.02 (-0.15, 0.11)	0.05 (-0.05, 0.16)
		1st trimester	0.19 (0.10, 0.27)	0.51	0.13 (-0.01, 0.27)	0.23 (0.12, 0.34)
	$PM_{2.5}$	2nd trimester	0.02 (-0.04, 0.07)	0.12	-0.05 (-0.14, 0.05)	0.06 (-0.01, 0.13)
		1st trimester	0.13 (0.07, 0.19)	0.23	0.06 (-0.05, 0.17)	0.17 (0.10, 0.25)
	$PM_{2.5}$ absorbance	2nd trimester	-0.11 (-0.21, -0.01)	0.51	-0.14 (-0.3, 0.03)	-0.09 (-0.22, 0.04)
		1st trimester	0.03 (-0.10, 0.16)	0.74	-0.01 (-0.21, 0.20)	0.05 (-0.12, 0.22)
	NO_2	2nd trimester	-0.09 (-0.14, -0.05)	0.43	-0.11 (-0.18, -0.04)	-0.09 (-0.14, -0.03)
		1st trimester	-0.09 (-0.15, -0.04)	0.64	-0.11 (-0.20, -0.01)	-0.09 (-0.16, -0.02)
	NO_x	2nd trimester	-0.08 (-0.12, -0.03)	0.09	-0.13 (-0.21, -0.05)	-0.04 (-0.10, 0.02)
		1st trimester	-0.05 (-0.10, 0.01)	0.28	-0.09 (-0.19, 0.01)	-0.02 (-0.09, 0.05)
Subscapular skinfolds			N=5,302		N=2,247	N=3,055
	PM_{10}	2nd trimester	0.12 (0.03, 0.20)	0.35	0.08 (-0.04, 0.21)	0.16 (0.05, 0.27)
		1st trimester	0.11 (0.02, 0.20)	0.37	0.05 (-0.09, 0.19)	0.17 (0.05, 0.28)
	$PM_{2.5}$	2nd trimester	0.09 (0.03, 0.15)	0.14	0.04 (-0.06, 0.14)	0.13 (0.05, 0.20)
		1st trimester	0.08 (0.02, 0.14)	0.09	-0.01 (-0.12, 0.10)	0.14 (0.06, 0.22)
	$PM_{2.5}$ absorbance	2nd trimester	0.05 (-0.05, 0.16)	0.71	0.06 (-0.11, 0.22)	0.05 (-0.08, 0.19)
		1st trimester	0.04 (-0.09, 0.17)	0.82	0.02 (-0.19, 0.22)	0.05 (-0.12, 0.23)
	NO_2	2nd trimester	0.01 (-0.03, 0.06)	0.87	0.02 (-0.05, 0.10)	0.01 (-0.05, 0.07)
		1st trimester	0.01 (-0.05, 0.06)	0.79	0.00 (-0.10, 0.09)	0.01 (-0.06, 0.08)
	NO_x	2nd trimester	0.02 (-0.03, 0.07)	0.58	0.02 (-0.07, 0.10)	0.03 (-0.03, 0.09)
		1st trimester	0.00 (-0.06, 0.06)	0.50	-0.02 (-0.12, 0.07)	0.02 (-0.06, 0.09)

[a]Birth weight and head circumference models adjusted for: gestational age (complete weeks and days) and its square, maternal age (years), parity (0, 1, 2+), socioeconomic position (maternal education and house tenure), maternal height, maternal weight at booking, maternal active tobacco smoking during pregnancy (yes, no), season of conception (cold, warm) and sex. Triceps and subscapular skinfolds models were further adjusted for maternal 2-hour post-load glucose at 26-28 weeks. Full population models were adjusted also for ethnicity (White British, Pakistani origin).

[b]Effect estimates correspond to 10 μg/m^3 increase in PM_{10}, 5 μg/m^3 increase in $PM_{2.5}$, 1 10^{-5}/m increase in $PM_{2.5}$ absorbance, 10 μg/m^3 increase in NO_2 and 20 μg/m^3 increase in NO_x. [c]Interaction between the indicated air pollutant and maternal ethnicity.

Table S5. Term babies (N=8,586) - Adjusted [a] models coefficients (95% CI) for air pollution exposure [b] and birth weight (g) head circumference (cm), triceps and subscapular skinfolds thickness (mm).

Outcome	Air pollutant	Pregnancy period	Full population	p-value of int.[c]	White British	Pakistani origin
Birth Weight			N=7,545		N=3,389	N=4,156
	PM_{10}	full pregnancy	-5 (-38, 28)	0.18	-34 (-82, 14)	29 (-16, 73)
		3rd trimester	-11 (-41, 19)	0.19	-40 (-85, 5)	19 (-22, 59)
	$PM_{2.5}$	full pregnancy	-6 (-29, 17)	0.03	-42 (-80, -5)	21 (-8, 50)
		3rd trimester	-10 (-31, 12)	0.04	-44 (-79, -10)	15 (-13, 42)
	$PM_{2.5}$ absorbance	full pregnancy	-6 (-56, 43)	0.01	-72 (-146, 2)	52 (-15, 119)
		3rd trimester	-8 (-48, 32)	0.16	-48 (-109, 12)	24 (-30, 77)
	NO_2	full pregnancy	5 (-20, 30)	0.04	-21 (-60, 18)	22 (-11, 55)
		3rd trimester	0 (-18, 18)	0.16	-18 (-46, 10)	12 (-12, 36)
	NO_x	full pregnancy	-11 (-34, 13)	0.07	-35 (-72, 2)	7 (-23, 37)
		3rd trimester	-9 (-28, 10)	0.18	-27 (-57, 2)	4 (-20, 29)
Head circumference			N=6,976		N=3,124	N=3,852
	PM_{10}	full pregnancy	-0.48 (-0.70, -0.27)	0.49	-0.56 (-0.87, -0.25)	-0.4 (-0.69, -0.11)
		3rd trimester	-0.19 (-0.28, -0.09)	0.02	-0.28 (-0.42, -0.13)	-0.11 (-0.24, 0.03)
	$PM_{2.5}$	full pregnancy	-0.34 (-0.49, -0.19)	0.05	-0.53 (-0.77, -0.29)	-0.20 (-0.39, -0.01)
		3rd trimester	-0.13 (-0.20, -0.06)	0.00	-0.26 (-0.37, -0.14)	-0.05 (-0.14, 0.04)
	$PM_{2.5}$ absorbance	full pregnancy	-0.46 (-1.26, 0.33)	0.12	-0.99 (-2.18, 0.21)	-0.02 (-1.10, 1.05)
		3rd trimester	-0.03 (-0.16, 0.10)	0.03	-0.14 (-0.34, 0.05)	0.05 (-0.12, 0.23)
	NO_2	full pregnancy	0.00 (-0.01, 0.01)	0.78	0.00 (-0.01, 0.01)	0.00 (-0.01, 0.01)
		3rd trimester	0.05 (-0.01, 0.11)	0.19	0.03 (-0.06, 0.12)	0.06 (-0.01, 0.14)
	NO_x	full pregnancy	-0.04 (-0.08, 0.00)	0.85	-0.02 (-0.08, 0.04)	-0.04 (-0.09, 0.00)
		3rd trimester	-0.01 (-0.07, 0.05)	0.45	-0.01 (-0.10, 0.09)	-0.01 (-0.09, 0.06)

Outcome	Air pollutant	Pregnancy period	Full population	p-value of int.[c]	White British	Pakistani origin
Triceps skinfolds			N=5,157		N=2,175	N=2,982
	PM_{10}	full pregnancy	0.17 (0.06, 0.28)	0.20	0.07 (-0.10, 0.25)	0.26 (0.11, 0.40)
		3rd trimester	0.15 (0.05, 0.25)	0.20	0.05 (-0.12, 0.21)	0.23 (0.10, 0.36)
	$PM_{2.5}$	full pregnancy	0.12 (0.05, 0.20)	0.05	0.01 (-0.13, 0.14)	0.19 (0.10, 0.29)
		3rd trimester	0.11 (0.04, 0.18)	0.07	0.00 (-0.13, 0.12)	0.18 (0.09, 0.26)
	$PM_{2.5}$ absorbance	full pregnancy	-0.06 (-0.22, 0.09)	0.39	-0.14 (-0.38, 0.11)	-0.01 (-0.21, 0.19)
		3rd trimester	0.02 (-0.11, 0.14)	0.32	-0.08 (-0.29, 0.12)	0.08 (-0.08, 0.24)
	NO_2	full pregnancy	-0.19 (-0.27, -0.12)	0.35	-0.23 (-0.36, -0.10)	-0.18 (-0.28, -0.08)
		3rd trimester	-0.06 (-0.11, 0.00)	0.44	-0.09 (-0.18, 0.00)	-0.04 (-0.11, 0.03)
	NO_x	full pregnancy	-0.10 (-0.17, -0.03)	0.04	-0.2 (-0.32, -0.07)	-0.04 (-0.13, 0.05)
		3rd trimester	-0.03 (-0.08, 0.03)	0.14	-0.09 (-0.19, 0.00)	0.02 (-0.06, 0.09)
Subscapular skinfolds			N=5,143		N=2,168	N=2,975
	PM_{10}	full pregnancy	0.22 (0.11, 0.33)	0.19	0.13 (-0.04, 0.31)	0.31 (0.16, 0.45)
		3rd trimester	0.22 (0.12, 0.33)	0.27	0.14 (-0.02, 0.31)	0.29 (0.15, 0.42)
	$PM_{2.5}$	full pregnancy	0.16 (0.09, 0.24)	0.04	0.05 (-0.08, 0.18)	0.23 (0.14, 0.33)
		3rd trimester	0.17 (0.09, 0.24)	0.11	0.07 (-0.05, 0.19)	0.22 (0.13, 0.31)
	$PM_{2.5}$ absorbance	full pregnancy	0.08 (-0.07, 0.24)	0.41	0.03 (-0.22, 0.28)	0.12 (-0.09, 0.33)
		3rd trimester	0.05 (-0.08, 0.17)	0.30	-0.05 (-0.25, 0.15)	0.10 (-0.06, 0.27)
	NO_2	full pregnancy	0.00 (-0.08, 0.08)	0.63	-0.01 (-0.14, 0.12)	0.00 (-0.11, 0.10)
		3rd trimester	-0.03 (-0.08, 0.03)	0.69	-0.04 (-0.13, 0.05)	-0.02 (-0.09, 0.05)
	NO_x	full pregnancy	0.02 (-0.06, 0.09)	0.34	-0.02 (-0.15, 0.11)	0.04 (-0.06, 0.13)
		3rd trimester	0.00 (-0.06, 0.05)	0.46	-0.04 (-0.13, 0.06)	0.01 (-0.06, 0.09)

[a] Birth weight and head circumference models adjusted for: gestational age (complete weeks and days) and its square, maternal age (years), parity (0, 1, 2+), socioeconomic position (maternal education and house tenure), maternal height, maternal weight at booking, maternal active tobacco smoking during pregnancy (yes, no), season of conception (cold, warm) and sex. Triceps and subscapular skinfolds models were further adjusted for maternal 2-hour post-load glucose at 26-28 weeks. Full population models were adjusted also for ethnicity (White British, Pakistani origin). [b]Effect estimates correspond to 10 $\mu g/m^3$ increase in PM_{10}, 5 $\mu g/m^3$ increase in $PM_{2.5}$, 1 $10^{-5}/m$ increase in $PM_{2.5}$ absorbance, 10 $\mu g/m^3$ increase in NO_2 and 20 $\mu g/m^3$ increase in NO_x. [c]Interaction between the indicated air pollutant and maternal ethnicity.

Table S6. Mothers who don't change residence during pregnancy (N=8,521) - Adjusted[a] models coefficients (95% CI) for ambient air pollution exposure[b] and birth weight (g), head circumference (cm), triceps and subscapular skinfolds thickness (mm).

Outcome	Air pollutant	Pregnancy period	Full population	p-value of int.[c]	White British	Pakistani origin
Birth weight			N=7,501		N=3,329	N=4,172
	PM_{10}	full pregnancy	-12 (-44, 21)	0.35	-44 (-93, 5)	23 (-21, 67)
		3rd trimester	-16 (-46, 14)	0.23	-43 (-87, 2)	9 (-31, 49)
	$PM_{2.5}$	full pregnancy	-14 (-37, 9)	0.46	-50 (-88, -12)	11 (-18, 40)
		3rd trimester	-16 (-37, 5)	0.28	-45 (-80, -11)	3 (-24, 30)
	$PM_{2.5}$ absorbance	full pregnancy	-6 (-56, 44)	0.05	-66 (-141, 9)	43 (-24, 110)
		3rd trimester	-8 (-49, 32)	0.42	-37 (-99, 25)	13 (-40, 66)
	NO_2	full pregnancy	9 (-16, 34)	0.08	-15 (-54, 24)	22 (-10, 55)
		3rd trimester	5 (-14, 23)	0.35	-9 (-37, 20)	12 (-12, 35)
	NO_x	full pregnancy	-4 (-27, 19)	0.15	-28 (-65, 8)	11 (-19, 41)
		3rd trimester	-2 (-20, 17)	0.38	-15 (-45, 14)	8 (-16, 32)
Head circumference			N=6,880		N=3,043	N=3,837
	PM_{10}	full pregnancy	-0.26 (-0.37, -0.16)	0.49	-0.30 (-0.45, -0.14)	-0.23 (-0.37, -0.08)
		3rd trimester	-0.22 (-0.32, -0.12)	0.04	-0.30 (-0.45, -0.15)	-0.15 (-0.28, -0.02)
	$PM_{2.5}$	full pregnancy	-0.20 (-0.28, -0.13)	0.06	-0.29 (-0.41, -0.17)	-0.14 (-0.24, -0.05)
		3rd trimester	-0.17 (-0.24, -0.10)	<0.001	-0.28 (-0.39, -0.16)	-0.10 (-0.19, -0.01)
	$PM_{2.5}$ absorbance	full pregnancy	-0.16 (-0.32, 0.00)	0.20	-0.23 (-0.48, 0.01)	-0.09 (-0.30, 0.12)
		3rd trimester	-0.09 (-0.22, 0.04)	0.07	-0.19 (-0.39, 0.02)	-0.03 (-0.20, 0.14)
	NO_2	full pregnancy	-0.01 (-0.09, 0.07)	0.87	0.00 (-0.12, 0.12)	-0.02 (-0.12, 0.08)
		3rd trimester	0.04 (-0.02, 0.10)	0.37	0.03 (-0.06, 0.12)	0.04 (-0.03, 0.12)
	NO_x	full pregnancy	-0.07 (-0.15, 0.00)	0.92	-0.05 (-0.17, 0.07)	-0.08 (-0.18, 0.01)
		3rd trimester	-0.01 (-0.07, 0.05)	0.64	0.00 (-0.10, 0.09)	-0.02 (-0.10, 0.06)

Outcome	Air pollutant	Pregnancy period	Full population	p-value of int.[c]	White British	Pakistani origin
Triceps skinfolds			N=4,996		N=2,074	N=2,922
	PM_{10}	full pregnancy	0.16 (0.04, 0.27)	0.13	0.05 (-0.13, 0.22)	0.25 (0.11, 0.40)
		3rd trimester	0.13 (0.02, 0.23)	0.17	0.01 (-0.15, 0.18)	0.21 (0.08, 0.35)
	$PM_{2.5}$	full pregnancy	0.10 (0.03, 0.18)	0.03	-0.03 (-0.16, 0.11)	0.18 (0.09, 0.28)
		3rd trimester	0.09 (0.02, 0.16)	0.05	-0.04 (-0.17, 0.08)	0.16 (0.07, 0.25)
	$PM_{2.5}$ absorbance	full pregnancy	-0.07 (-0.22, 0.09)	0.16	-0.20 (-0.46, 0.06)	0.02 (-0.18, 0.22)
		3rd trimester	0.03 (-0.10, 0.15)	0.42	-0.08 (-0.29, 0.13)	0.09 (-0.08, 0.25)
	NO_2	full pregnancy	-0.19 (-0.26, -0.11)	0.39	-0.22 (-0.35, -0.09)	-0.17 (-0.27, -0.08)
		3rd trimester	-0.05 (-0.10, 0.01)	0.72	-0.07 (-0.16, 0.02)	-0.04 (-0.11, 0.03)
	NO_x	full pregnancy	-0.09 (-0.17, -0.02)	0.04	-0.20 (-0.33, -0.07)	-0.04 (-0.13, 0.05)
		3rd trimester	-0.02 (-0.08, 0.04)	0.24	-0.08 (-0.18, 0.02)	0.02 (-0.06, 0.09)
Subscapular skinfolds			N=4,983		N=2,067	N=2,916
	PM_{10}	full pregnancy	0.19 (0.08, 0.30)	0.18	0.10 (-0.07, 0.28)	0.27 (0.12, 0.42)
		3rd trimester	0.20 (0.10, 0.31)	0.29	0.13 (-0.04, 0.29)	0.26 (0.12, 0.40)
	$PM_{2.5}$	full pregnancy	0.14 (0.06, 0.21)	0.04	0.02 (-0.11, 0.16)	0.20 (0.11, 0.30)
		3rd trimester	0.14 (0.07, 0.22)	0.12	0.05 (-0.07, 0.18)	0.20 (0.10, 0.29)
	$PM_{2.5}$ absorbance	full pregnancy	0.08 (-0.08, 0.24)	0.33	0.01 (-0.25, 0.26)	0.12 (-0.09, 0.33)
		3rd trimester	0.07 (-0.06, 0.20)	0.55	0.01 (-0.20, 0.22)	0.10 (-0.07, 0.27)
	NO_2	full pregnancy	0.00 (-0.08, 0.08)	0.87	0.01 (-0.12, 0.14)	-0.01 (-0.12, 0.09)
		3rd trimester	-0.01 (-0.07, 0.05)	0.87	0.00 (-0.09, 0.09)	-0.02 (-0.09, 0.05)
	NO_x	full pregnancy	0.00 (-0.07, 0.08)	0.43	-0.02 (-0.15, 0.10)	0.02 (-0.08, 0.12)
		3rd trimester	0.00 (-0.06, 0.06)	0.79	-0.01 (-0.11, 0.09)	0.01 (-0.07, 0.08)

[a]Birth weight and head circumference models adjusted for: gestational age (complete weeks and days) and its square, maternal age (years), parity (0, 1, 2+), socioeconomic position (maternal education and house tenure), maternal height, maternal weight at booking, maternal active tobacco smoking during pregnancy (yes, no), season of conception (cold, warm) and sex. Triceps and subscapular skinfolds models were further adjusted for maternal 2-hour post-load glucose at 26-28 weeks. Full population models were adjusted also for ethnicity (White British, Pakistani origin).

[b]Effect estimates correspond to 10 μg/m^3 increase in PM_{10}, 5 μg/m^3 increase in $PM_{2.5}$, 1 10^{-5}/m increase in $PM_{2.5}$ absorbance, 10 μg/m^3 increase in NO_2 and 20 μg/m^3 increase in NO_x. [c]p-Value for interaction between the indicated air pollutant and maternal ethnicity.

Table S7. Unemployed (n=4,436) vs. employed (n=3,201) mothers - Adjusted[a] models coefficients (95% CI) for air pollution exposure[b] and birth weight (g) head circumference (cm) triceps and subscapular skinfolds thickness (mm).

Outcome	Air pollutant	Pregnancy period	Unemployed	Employed	p-value of int.[c]
Birth weight			N=4,063	N=2,281	
	PM_{10}	full pregnancy	27 (-25, 79)	-26 (-95, 43)	0.18
		3rd trimester	12 (-36, 60)	-46 (-108, 16)	0.27
	$PM_{2.5}$	full pregnancy	8 (-28, 44)	-15 (-63, 37)	0.12
		3rd trimester	1 (-32, 34)	-28 (-72, 16)	0.16
	$PM_{2.5}$ absorbance	full pregnancy	40 (-28, 108)	-82 (-172, 8)	0.03
		3rd trimester	20 (-35, 76)	-47 (-118, 24)	0.23
	NO_2	full pregnancy	10 (-23, 44)	8 (-37, 52)	0.54
		3rd trimester	-2 (-26, 22)	12 (-20, 43)	0.84
	NO_x	full pregnancy	7 (-24, 38)	-27 (-70, 16)	0.11
		3rd trimester	1 (-24, 26)	-11 (-45, 23)	0.47
Head circumference			N=3,749	N=2,102	
	PM_{10}	full pregnancy	-0.21 (-0.38, -0.04)	-0.36 (-0.59, -0.12)	0.09
		3rd trimester	-0.16 (-0.32, -0.01)	-0.33 (-0.54, -0.11)	0.05
	$PM_{2.5}$	full pregnancy	-0.14 (-0.25, -0.02)	-0.25 (-0.42, -0.09)	0.09
		3rd trimester	-0.11 (-0.21, 0.00)	-0.24 (-0.39, -0.08)	0.04
	$PM_{2.5}$ absorbance	full pregnancy	-0.09 (-0.31, 0.12)	-0.18 (-0.49, 0.12)	0.34
		3rd trimester	0.01 (-0.17, 0.18)	-0.06 (-0.30, 0.19)	0.28
	NO_2	full pregnancy	-0.01 (-0.12, 0.09)	0.03 (-0.12, 0.18)	1.00
		3rd trimester	0.05 (-0.03, 0.12)	0.06 (-0.04, 0.17)	0.67
	NO_x	full pregnancy	-0.07 (-0.17, 0.03)	-0.09 (-0.24, 0.05)	0.48
		3rd trimester	0.01 (-0.07, 0.09)	-0.03 (-0.14, 0.09)	0.33

Outcome	Air pollutant	Pregnancy period	Unemployed	Employed	p-value of int.[c]
Triceps skinfolds			N=2,904	N=1,943	
	PM_{10}	full pregnancy	0.34 (0.18, 0.49)	0.10 (-0.15, 0.36)	0.06
		3rd trimester	0.29 (0.14, 0.44)	0.10 (-0.13, 0.34)	0.28
	$PM_{2.5}$	full pregnancy	0.46 (0.24, 0.68)	0.01 (-0.19, 0.20)	0.05
		3rd trimester	0.41 (0.21, 0.62)	0.03 (-0.15, 0.21)	0.20
	$PM_{2.5}$ absorbance	full pregnancy	0.99 (-1.02, 3.00)	-0.63 (-3.91, 2.64)	0.08
		3rd trimester	1.30 (-0.37, 2.97)	0.50 (-2.24, 3.24)	0.50
	NO_2	full pregnancy	-0.16 (-0.26, -0.06)	-0.25 (-0.42, -0.09)	0.66
		3rd trimester	-0.05 (-0.12, 0.02)	-0.09 (-0.21, 0.03)	0.76
	NO_x	full pregnancy	-0.03 (-0.07, 0.02)	-0.13 (-0.21, -0.04)	0.20
		3rd trimester	0.00 (-0.04, 0.04)	-0.05 (-0.12, 0.02)	0.80
Subscapular skinfolds			N=2,898	N=1,937	
	PM_{10}	full pregnancy	0.25 (0.08, 0.41)	-0.08 (-0.28, 0.13)	0.06
		3rd trimester	0.23 (0.08, 0.39)	-0.01 (-0.2, 0.18)	0.52
	$PM_{2.5}$	full pregnancy	0.18 (0.07, 0.29)	-0.02 (-0.17, 0.12)	0.12
		3rd trimester	0.17 (0.07, 0.28)	0.02 (-0.12, 0.15)	0.67
	$PM_{2.5}$ absorbance	full pregnancy	0.25 (0.04, 0.45)	-0.11 (-0.37, 0.15)	0.15
		3rd trimester	0.07 (-0.1, 0.24)	-0.09 (-0.31, 0.13)	0.92
	NO_2	full pregnancy	0.02 (-0.08, 0.12)	-0.03 (-0.16, 0.1)	0.97
		3rd trimester	-0.06 (-0.13, 0.02)	0.02 (-0.08, 0.11)	0.09
	NO_x	full pregnancy	0.03 (-0.07, 0.12)	-0.05 (-0.18, 0.08)	0.81
		3rd trimester	-0.05 (-0.12, 0.03)	0.01 (-0.09, 0.11)	0.13

[a]Birth weight and head circumference models adjusted for: gestational age (complete weeks and days) and its square, maternal age (years), parity (0, 1, 2+), socioeconomic position (maternal education and house tenure), maternal height, maternal weight at booking, maternal active tobacco smoking during pregnancy (yes, no), season of conception (cold, warm) and sex. Triceps and subscapular skinfolds models were further adjusted for maternal 2-hour post-load glucose at 26-28 weeks. Full population models were adjusted also for ethnicity (White British, Pakistani origin).

[b]Effect estimates correspond to 10 μg/m^3 increase in PM_{10}, 5 μg/m^3 increase in $PM_{2.5}$, 1 10^{-5}/m increase in $PM_{2.5}$ absorbance, 10 μg/m^3 increase in NO_2 and 20 μg/m^3 increase in NO_x. [c]p-Value for interaction between the indicated air pollutant and employment status (yes /no).

Table S8. Non-smokers (N=7,005) vs smoker (N=1,645) mothers - Adjusted[a] models coefficients (95% CI) for air pollution exposure[b] and birth weight (g), head circumference (cm), triceps and subscapular skinfolds thickness (mm).

Outcome	Air pollutant	Pregnancy period	Non-Smokers	Smokers	p-value of int.[c]
Birth weight			N=6,447	N=1,522	
	PM_{10}	full pregnancy	-18 (-54, 17)	39 (-33, 110)	0.10
		3rd trimester	-20 (-52, 13)	22 (-44, 87)	0.57
	$PM_{2.5}$	full pregnancy	-14 (-39, 10)	15 (-38, 69)	0.05
		3rd trimester	-15 (-8, 8)	6 (-43, 55)	0.36
	$PM_{2.5}$ absorbance	full pregnancy	-3 (-56, 51)	-3 (-114, 108)	0.86
		3rd trimester	-4 (-47, 39)	-27 (-120, 67)	0.40
	NO_2	full pregnancy	13 (-14, 40)	-6 (-64, 52)	0.77
		3rd trimester	9 (-10, 29)	-19 (-62, 23)	0.16
	NO_x	full pregnancy	-4 (-29, 21)	-3 (-57, 51)	0.62
		3rd trimester	0 (-20, 19)	-10 (-53, 33)	0.60
Head circumference			N=5,920	N=1,410	
	PM_{10}	full pregnancy	-0.29 (-0.41, -0.17)	-0.20 (-0.43, 0.03)	0.42
		3rd trimester	-0.22 (-0.33, -0.11)	-0.22 (-0.43, -0.01)	0.60
	$PM_{2.5}$	full pregnancy	-0.2 (-0.28, -0.11)	-0.18 (-0.35, -0.01)	0.55
		3rd trimester	-0.15 (-0.22, -0.07)	-0.19 (-0.34, -0.03)	0.51
	$PM_{2.5}$ absorbance	full pregnancy	-0.14 (-0.31, 0.04)	-0.07 (-0.43, 0.28)	0.73
		3rd trimester	-0.05 (-0.19, 0.09)	-0.07 (-0.36, 0.23)	0.42
	NO_2	full pregnancy	-0.02 (-0.10, 0.07)	0.04 (-0.15, 0.22)	0.60
		3rd trimester	0.05 (-0.01, 0.11)	0.05 (-0.08, 0.18)	0.52
	NO_x	full pregnancy	-0.10 (-0.18, -0.01)	0.03 (-0.15, 0.20)	0.24
		3rd trimester	-0.02 (-0.09, 0.04)	0.06 (-0.08, 0.19)	0.80

Outcome	Air pollutant	Pregnancy period	Non-Smokers	Smokers	p-value of int.[c]
Triceps skinfolds			N=4,378	N=938	
	PM_{10}	full pregnancy	0.15 (0.03, 0.27)	0.18 (-0.07, 0.43)	0.99
		3rd trimester	0.14 (0.03, 0.25)	0.13 (-0.10, 0.36)	0.53
	$PM_{2.5}$	full pregnancy	0.10 (0.02, 0.18)	0.15 (-0.02, 0.33)	0.70
		3rd trimester	0.10 (0.02, 0.18)	0.12 (-0.05, 0.28)	0.36
	$PM_{2.5}$ absorbance	full pregnancy	-0.06 (-0.22, 0.11)	-0.07 (-0.44, 0.29)	0.99
		3rd trimester	0.03 (-0.11, 0.16)	0.01 (-0.29, 0.32)	0.37
	NO_2	full pregnancy	-0.19 (-0.27, -0.1)	-0.18 (-0.37, 0.01)	0.95
		3rd trimester	-0.05 (-0.11, 0.01)	-0.04 (-0.17, 0.09)	0.29
	NO_x	full pregnancy	-0.1 (-0.18, -0.02)	-0.06 (-0.25, 0.13)	0.71
		3rd trimester	-0.03 (-0.09, 0.03)	0.01 (-0.13, 0.16)	0.23
Subscapular skinfolds			N=4,365	N=937	
	PM_{10}	full pregnancy	0.17 (0.05, 0.29)	0.36 (0.11, 0.61)	0.36
		3rd trimester	0.18 (0.07, 0.30)	0.34 (0.11, 0.57)	0.13
	$PM_{2.5}$	full pregnancy	0.13 (0.04, 0.21)	0.27 (0.09, 0.45)	0.26
		3rd trimester	0.14 (0.06, 0.21)	0.26 (0.09, 0.42)	0.09
	$PM_{2.5}$ absorbance	full pregnancy	0.05 (-0.12, 0.22)	0.25 (-0.11, 0.62)	0.42
		3rd trimester	0.05 (-0.09, 0.19)	0.06 (-0.25, 0.37)	0.46
	NO_2	full pregnancy	-0.01 (-0.1, 0.07)	0.08 (-0.11, 0.28)	0.36
		3rd trimester	-0.01 (-0.07, 0.05)	-0.05 (-0.18, 0.09)	0.58
	NO_x	full pregnancy	-0.02 (-0.1, 0.06)	0.21 (0.02, 0.40)	0.04
		3rd trimester	-0.01 (-0.07, 0.05)	0.03 (-0.12, 0.18)	0.27

[a] Birth weight and head circumference models adjusted for: gestational age (complete weeks and days) and its square, maternal age (years), parity (0, 1, 2+), socioeconomic position (maternal education and house tenure), maternal height, maternal weight at booking, season of conception (cold, warm) and sex. Triceps and subscapular skinfolds models were further adjusted for maternal 2-hour post-load glucose at 26-28 weeks. Full population models were adjusted also for ethnicity (White British, Pakistani origin). [b]Effect estimates correspond to 10 $\mu g/m^3$ increase in PM_{10}, 5

μg/m^3 increase in $PM_{2.5}$, 1 10^{-5}/m increase in $PM_{2.5}$ absorbance, 10 μg/m^3 increase in NO_2 and 20 μg/m^3 increase in NO_x. [c]Interaction between the indicated air pollutant and maternal ethnicity
